# “Unchain My Heart.” A Defense Industrial Policy Agenda for Germany’s *Zeitenwende*

**DOI:** 10.1007/s12399-022-00926-4

**Published:** 2022-10-18

**Authors:** Heiko Borchert, Torben Schütz, Joseph Verbovszky

**Affiliations:** Defense AI Observatory, Helmut Schmidt University/University of the Armed Forces, Hamburg, Holstenhofweg 85, 22043 Hamburg, Germany

**Keywords:** Defense industrial policy, Innovation, Geoeconomics, Risk appetite, Security of supply, Rüstungsindustriepolitik, Innovation, Geoökonomie, Risikoappetit, Versorgungssicherheit

## Abstract

A *Zeitenwende* that delivers one of Europe’s most powerful armed forces needs to correct the (non-existent) position of defense industrial issues within German political discourse. This paper therefore develops a defense industrial policy agenda based on four lines of effort: Make daringness Germany’s prime ambition; establish Germany as a defense industrial framework nation; promote German defense industrial diplomacy with foreign partners; solidify a true public-private defense partnership between the German government and the defense industrial base.

## Abstract (de)

Soll die Zeitenwende-Politik die Bundeswehr zu einer der schlagkräftigsten Streitkräfte Europas machen, muss sie den Umstand korrigieren, dass rüstungsindustriepolitische Aspekte im deutschen politischen Diskurs kaum eine Rolle spielen. Der Beitrag entwickelt eine rüstungsindustriepolitische Agenda basierend auf Wagemut als strategischer Ambition, der rüstungsindustriellen Rahmennation als Ordnungsansatz, rüstungsindustriellen Beziehungen als diplomatischem Instrument und einer echten strategischen Partnerschaft mit der Rüstungsindustrie.

## Introduction

German foreign and security policy currently treats defense industrial issues with benign neglect, creating a social comfort zone that prevents defense industrial issues from interfering with everyday political life. This attitude is deep-seated within the political elite and has evolved over decades as a combination of lesson’s learned from the country’s past.

This paper argues that Germany’s political establishment must actively engage defense industrial issues as strategic tools of foreign and security policy or risk the € 100 billion *Sondervermögen* (special fund) becoming a gigantic “fire and forget alimentation”. Paraphrasing Joe Coker, the defense industry is at the heart of Germany’s security architecture and is constitutionally enshrined. The German basic law sets out the establishment of armed forces. Armed forces require capabilities, and defense companies are paramount in providing and developing the industrial base necessary to produce the defense solutions that underpin these capabilities. Currently, however, defense companies have been caught in a straitjacket since different defense industrial policy instruments are mutually blocking rather than mutually reinforcing.

Unchaining Germany’s defense industry is difficult. Political acceptance of the defense industry as a source of national security, innovation, and economic well-being needs to increase. Because of Russia’s war of aggression in Ukraine attitudes are changing among leaders of the coalition government but persistently driving normative change is likely to remain inconsistent. Second, the Bundeswehr’s underfunding is a result of Germany’s normative preferences. Normative conformity, rather than capability output shape the requirements of the German defense industrial base. Thus, more money won’t solve any challenges if spent in a way that rewards compliance with the status quo at the cost of risk-driven performance. Finally, limited prospects at home drive business to foreign markets, but foreign countries’ interests in transfer of technology risk undercutting the technological edge at home. At the same time export rules make foreign market access a question of political acceptance in Germany rather than the result of superior corporate strategy and products.

Consequently, the German government needs bold moves to make the *Zeitenwende* a meaningful defense policy paradigm.^1^ Change needs to come from the top with the Bundestag playing a more vigorous role in demanding performance driven defense innovation. In so doing, promoting daringness to take risk, also financial risk, will be essential. In parallel a new defense industrial coordinator of the government could streamline responsibilities currently scattered among too many actors. Germany also needs a proper defense industrial policy to readjust the national defense ecosystem, establish Germany as a defense industrial framework nation, put forward the idea of supply webs and defense industrial diplomacy and solidify a strategic-level public-private defense partnership.

This paper proceeds in three steps. It begins with a discussion in the context of German *Zeitenwende* of the expectations raised by Chancellor Scholz’ February 2022 speech and the need to spend the Bundeswehr special fund wisely and effectively; we argue that the current defense industrial policy framework is insufficient. The body and conclusion of the paper then discusses four lines of effort to develop a defense industrial policy blueprint that mirrors the ambition set out by the Chancellor: pushing back aggression and ensuring freedom of maneuver amid increasingly assertive strategic competitors.

## *Zeitenwende:* are you serious?

“We are experiencing a turning point” (Deutscher Bundestag 2022, 1350) said Chancellor Scholz in his speech at the extraordinary meeting of the German parliament on February 27, 2022. Turning point, or *Zeitenwende,* is meant to describe significant policy changes such as the increase of Germany’s defense budget to 2% of GDP, a one-time special fund of € 100 billion for defense procurement, and the aim to reduce Germany’s energy dependence on Russia. Two days prior, the government had decided to deliver weapons to Ukraine.

Germany is not alone in responding to the fundamental change in Europe’s security environment. Allied partners in NATO and EU readjust their policies, too. This broader perspective highlights four themes that create a unique opportunity for Berlin to restructure its defense industrial policy stance.

First, European states will spend significantly more money on defense than previously anticipated. Both the German special fund of € 100 billion and significant defense spending increases announced by other European allies – twelve at the time of this writing (Koenig 2022, 3) – will expand the European defense market. Current estimates are at around € 200 billion more “over the coming years” (Barigazzi 2022). Even if stretched over ten years, this amount would equal an increase in overall European defense spending of about 10%.

Second, European arsenals are changing rapidly. As EU and NATO allies transfer equipment to Ukraine, including from active service, demand for replacements increases (Oliemans & Mitzer 2022). Moreover, procurement modernization is likely to shape the agenda as only few countries have so far announced plans to modernize military structures and personnel. While Ukraine demands more military equipment support from EU and NATO nations, their material stockpiles are depleting, and industrial production capacity limits and shortfalls are opening. Capacity shortfalls, in turn, open doors for non-European defense companies from the United States, Israel, Turkey, and South Korea, for example, to broaden their footprint in European arsenals thereby effectively closing these market segments for European suppliers for at least a generation of weapon systems.

Third, corporate supply chains are increasingly perceived as fragile. Cooperation that leads to dependence has become toxic through the Covid-19 pandemic and the war in Ukraine. As we discuss, geoeconomics creates a new Achilles’ heel that existing defense ecosystems are ill-prepared to tackle.

Lastly, there are unique domestic windows of opportunity in Germany. Right now, work is ongoing on the first National Security Strategy and a new arms export law. As both capstone documents will have consequences for industry, an innovative defense industrial policy could leverage this work in progress. However, in both cases time is of the essence, as drafts are already in the making. The National Security Strategy is slated to be released in early 2023, whereas the timeline for the arms export law is unknown.

## Why the current defense industrial approach is insufficient

Today, Germany operates a light defense industrial policy framework. The 2016 Defense white paper (Bundesregierung 2016) identifies defense-relevant research and technology (R&T) as a key driver of defense innovation and the defense industry. It also emphasizes the need for the Bundeswehr to tap into civilian innovation ecosystems. The 2018 capstone document of the Bundeswehr (BMVg 2018) goes one step further and establishes support for defense-relevant core technologies as well as defense cooperation with international partners as an official task of the Bundeswehr. In 2020, the government published the latest iteration of its strategy paper on “strengthening the security and defense industries” which covers R&T, export support, and foreign direct investment control. Most importantly, this document updates the list of security/defense-relevant core technologies and describes the preferred ways to retain and procure them (Bundesregierung 2020). In parallel, the German government has stepped-up institutional efforts, for example, to coordinate inter-agency action with industrial activities to support defense exports.

We acknowledge the progress made by the German government, but the status quo does not hold up to the current challenges. These we identify as geoeconomics, foreign markets, the current ecosystem, funding, and competition over talent.

First, geoeconomics, or the use of economic instruments to achieve political goals (Blackwill and Harris 2016), presents a toxic cocktail of challenges for defense industrial policy. On the one hand, the defense industry requires key raw materials such as bauxit, cobalt, gallium or titanium originating from countries like Russia, China or sources in Africa or Latin America (European Commission 2020; Harris 2022). Dependence on few sources from these regions runs the risk of deliberate supply shortages that undercut Western defense production capacities. On the other hand, emerging technologies like robotic and autonomous systems, artificial intelligence, or space technologies are said to contribute to superior future battlefield effects. Many of these technologies originate outside the defense sector and are at the heart of today’s geoeconomic competition revolving around access to technologies, corporate supply chains, market access, and industry standards. Growing emphasis on the importance of these emerging technologies subjects the defense establishment to this new and unfamiliar geoeconomic dynamic (Borchert et al. 2022).

Second, foreign market dynamics reinforce the geoeconomic challenge. As Borchert and Thiele (2014) have argued, there is hardly any access to foreign markets without transfer and localization of knowledge and technology. Aspiring new defense players want to establish their own defense industrial and technology base to diversify away from traditional suppliers and gain more strategic leeway. Therefore, defense companies from Germany and other Western countries walk a thin line between transferring technology and expertise in return for market access while at the same time maintaining their own edge vis-à-vis future competitors. This becomes more challenging as the integration of emerging technologies can shorten development cycles and enable competitors to leapfrog. In response, new modes of public-private technology and skills road mapping are needed to better understand what can be shared with foreign partners and which red lines are needed to maintain superiority.

Third, the current German defense ecosystem consisting of the defense industry, R&T organizations (RTO), the Ministry of Defense (MoD) and the Bundeswehr is unbalanced. There are two major sources of this imbalance. On the one hand, commercially driven technology developments follow a different rationale, the respective products ground in different life cycles, and commercial companies scale at a pace unknown to traditional defense actors. This changes the power balance especially as digital companies entering the defense market attempt to emulate incumbent players by using digital platforms as a means to create monopolies and reorganize supplier networks. On the other hand, roles and responsibilities delineated among ecosystem partners are increasingly blurred. Defense authorities have partly outsourced tasks like defining defense capability requirements, auditing project performance, and independently testing and validating technologies to RTO and companies. Originally established to support the Bundeswehr and the MoD, these actors increasingly make their case with private sector partners thereby leveraging intimate knowledge gained from assisting defense authorities. By blending their role as “non-aligned”, custodians meant to advise the public sector with hard commercial interests, they undercut competition, proliferate knowledge in ways that can be detrimental to commercial proprietary information and harm the dynamic of the defense ecosystem.

Fourth, talking about a defense funding challenge amid the € 100 billion special fund may sound paradox. But two funding aspects are problematic. First, so-called environmental, social, and governance principles (ESG) increasingly shape public and private investments. While ESG definitions remain unclear in practice, these blurred definitions lead investors to increasingly consider defense companies a reputation risk. Second, private equity and venture capital create a dual funding challenge. So far, both have offered attractive financial perspectives. These have lured startup companies working on emerging technologies thereby drying up the landscape of attractive defense newcomers. Right now, rising interest rates increase corporate refinancing costs. At the very moment commercial companies working on emerging technologies become attractive for defense, it becomes highly uncertain who will fund them and who will survive amid rising interest rates.

Finally, and closely related to the funding challenge, the defense industry has a growing aging and talent problem. At least in the past, commercial technology champions working on emerging technologies did not shy away from paying several hundred thousand of euros a year as salaries plus company stocks to attract the best talents (Metz 2017). The public sector and the defense industry can hardly compete with these incentives. In addition, normative and societal preferences have made the defense industry an unattractive working place. Candidates for management jobs remain difficult to find “as nobody wants to reveal working for a company that produces weapons”, a German headhunter recently told *Handelsblatt,* the leading German business daily (Backovic 2022). Counterintuitively, the headhunter argued, the German government’s defense spending spree could make things worse as defense companies will compete more intensely for scarce talent given the need to ramp up efforts to fulfill the government’s growing defense orders. But if talents continue to remain on short supply, who is going to end up working on emerging technologies for defense and what quality of work will the defense community get in the future (Cummings 2018, p. 16)?

## Defense Industrial Policy for the *Zeitenwende*

In 2019, former minister of economic affairs Peter Altmaier launched the first ever German industrial policy. He said a heavier government hand was needed in response to China which excelled at concerted efforts to define, implement, and enforce its economic-industrial vision versus industrialized nations (BMWi 2019a, b). Altmaier argued that industrial policy that protects and advances industrial interests is justified “if a country’s market forces are insufficient to bolster its innovation and competitiveness” (BMWi 2019a, p. 2).

Defense, however, does not suffer from market failure, but rather policy failure. There is no defense market in a proper economic sense. Rather, governments define product specifications, set price and profit levels, separate wanted from unwanted suppliers, define which foreign partners are palatable or constitute inacceptable risks, shape R&T priorities, and might even hold stakes in companies (Borchert and Thiele 2014, p. 369). Defense is a sovereign task and so it falls on the government to define the defense industrial policy framework for industry to work properly.

Against this background, defense industrial policy starts with identifying the paradigm that shapes German defense industrial thinking and the strategic goals to be accomplished. The remainder of this paper illustrates four lines of effort to support the *Zeitenwende* (Tab. 1): make daringness Germany’s prime ambition; become a defense industrial framework nation; promote defense industrial diplomacy with foreign partners; and solidify a true public-private defense partnership.

**Table 1:** Four lines of effort to advance Germany’s defense industrial policy

**Make daringness Germany’s prime ambition** – Make the Bundestag a champion of defense daringness – Appoint a Defense Industrial Coordinator – Assess and streamline the current procurement framework – Use the Zivilklausel more flexibly – Adopt a new innovation management method – Advance defense sandboxing and strengthen defense test units – Establish a Bundeswehr Digital Commons
**Become a defense industrial framework nation** – Clarify what Germany wants to be – Set up a government-to-government framework – Advance reverse empowerment
**Promote defense industrial diplomacy with partners** – Define the task portfolio – Leverage German defense subsidiaries abroad as technology bridges to advance regional supply webs – Consider defense skills a strategic asset
**Solidify a true public-private partnership** – Clarify that the defense industry is ESG compatible – Engage in technology road mapping with industry – Recalibrate competition vs consolidation – Advance competitive intelligence

### Make daringness Germany’s prime ambition

Germany’s defense industrial policy needs to serve four essential goals. First, the Bundeswehr needs to be equipped adequately to perform its assigned political role as the mainstay of national and alliance defense. Second, Germany’s defense equipment must be interoperable with partners. Third, considering the rapid depletion of defense stockpiles amid Russia’s war in Ukraine and given increasingly assertive strategic competitors, defense relevant security of supply becomes paramount. Finally, defense innovation – defined as ability to deliver military added value by synchronizing conceptual, organizational, and technological transformation in line with operational experience and cultural predisposition (Borchert et al. 2021, p. 13) – needs to be unleashed. Implementing these goals requires a whole-of-lifecycle approach, that includes concept development and experimentation; R&T; product development; production; maintenance, repair, and overhaul; as well as end-of-life displacement. Ultimately, however, everything depends on the paradigm that shapes the definition of these goals.

Given the *Zeitenwende* rhetoric of repelling aggression and preparing for long and protracted strategic competition with rivals that operate along different worldviews, we contend that daringness must become Germany’s defense industrial policy paradigm. Daringness responds to the fact that risk aversion invites adversarial risk aggression (Matlary 2020, p. 79). Daringness is needed to tilt the prevailing defense industrial approach from its current focus on conformity with the status quo to a risk-informed focus on performance (Borchert et al. 2022, p. 17, 19). Daringness also creates fertile ground for risk-taking, benefitting from failure, and experimentation to shape tomorrow’s defense solutions.

Infusing daringness into the German defense establishment will take hard work and need to tackle cultural, organizational, and technological issues in parallel. We propose a seven-point agenda that includes a key role for the Bundestag, the appointment of a new defense industrial coordinator, the streamlining of the current procurement framework, a more flexible handling of the famous *Zivilklausel*, a real option-based approach to innovation management, strengthening defense sandboxing and military test units and the set-up of a new Bundeswehr Digital Commons under public control.

#### Make the Bundestag a champion of defense daringness

If daringness is to serve as a new paradigm for defense policy and defense industrial matters, it must have the full backing of the political establishment. The Bundestag can meaningfully support daringness by creating a new budget line and giving it a face (Schütz et al. 2022).

First, the Bundestag will need to become a place of informed exchange on defense and security issues, including and beyond the defense committee. Establishing advanced defense and security courses for parliament members (especially new ones) and their staffs help create an informed and educated political leadership, which can make defense and security decisions with confidence and ease.

Second, the Bundestag should expand the existing budget line on concept development and experimentation in the defense budget to cover innovation and experimentation. The respective budget should fund national and multinational demonstration and experimentation projects, rapid product prototyping, and a new series of competitions to assess the maturity of emerging concepts and technologies in view of future battlefield requirements.

Additionally, the Bundestag’s defense committee should appoint one of its members, for example the chairwoman or chairman, as defense innovation and experimentation rapporteur. Her or his job is devoted to empowering Bundeswehr transformation, ensure political support to cut back bureaucratic red tape, stimulate organizational transformation, and advance high-risk/high-benefit capability and technology development. Creating a face in the Bundestag drives home the important political message that parliamentarians care about change, daringness, and risk-taking. As a patron she or he would oversee the innovation and experimentation budget, invite companies and research institutes to innovation and experimentation exercises, and host hearings with national and international experts focusing on defense innovation.

#### Appoint a Defense Industrial Coordinator

Right now, responsibilities for defense industrial matters are scattered among the Chancellery, the MoD, the Ministry of Foreign Affairs and the Ministry for Economic Affairs and Climate Action. This set up renders defense industrial coordination cumbersome and gives veto actors maximum leeway. At the same time, however, the German government and individual ministers can appoint coordinators and commissioners with a special portfolio. There are, for example, government coordinators for maritime industry and tourism as well as aerospace policy. Both also deal with defense issues, but only as part of a broader portfolio.

In contrast, a government coordinator for the defense industry would focus exclusively on defense industrial matters in Germany and abroad. Giving the coordinator an international role is important in view of government-to-government support that we discuss later. In addition, the coordinator would primarily focus on developing and adjusting the defense technology and industrial base commensurate with geoeconomic challenges, future force requirements, and the long-term development plans of Germany’s defense companies and RTO. To benefit from non-defense companies, the coordinator would also play a key role in broadening the national ecosystem and advance networking between traditional and new players.

As defense industrial matters cross several policy domains and different technology sectors, assigning the new coordinator with the Chancellery’s Group 23 (formerly Group 22) seems appropriate as this could facilitate interaction with the envisaged national security coordination institutions that the new national security strategy is likely to establish.

#### Assess and streamline the current procurement framework

The Bundestag can further unleash the power of innovation by adjusting the prevailing procurement model. The so-called € 25 M ceiling is one aspect. Every project above this ceiling needs to be approved by the Bundestag before the MoD can sign the respective contracts. This approach is cumbersome. Therefore, the Bundestag should switch from a project-based approach to a capability-based approach that mirrors armed forces planning. The capability-based approach would entail procurement decisions based on functional capability packages, thereby moving the current procurement pipeline into a procurement portfolio that could be assessed based on the strategic relevance and necessity of the capabilities and the underlining technology maturity. This broader perspective also provides an option to drop the self-restraining ceiling altogether and open doors for multi-year, rather than annual funding cycles.

Over the past couple of weeks, the Bundestag has initiated legislation to fast-track procurement (Bundestag 2022). This approach should be expanded to include the civilian admission criteria Bundeswehr material needs to fulfill to get certified for use. The Bundestag’s defense committee should ask the MoD to audit these criteria with the goal to cut back their number to an essential minimum and thus fast-track the commissioning of new defense equipment.

#### Use the Zivilklausel more flexibly

The civil clause (*Zivilklausel*) is a German peculiarity; it is a voluntary self-declaration that prevents universities from engaging in defense research and cooperating with the defense industry. The clause originated at the University of Bremen in 1986 and experienced an expansion to other German universities after the end of the Cold War. Right now, more than 70 universities and universities of applied sciences have signed up to the civil clause.^2^

The civil clause is a cause of contention. It is out of tune with the current geostrategic reality in Europe and effectively splits the German academic ecosystem into defense and non-defense parts thus depriving each part of the benefits of learning from the other. While a full-scale abolishment of the civil clause is not in the cards today, universities complying with the clause should consider rendering their practice more flexible by delegating the authority to decide to single institutes. Each institute willing and able to work on defense issues could be asked to conduct an ex-ante assessment of the ethical, legal, and societal consequences of the respective project. In addition, the MoD and the institute’s partners would contribute to the assessment by describing the strategic relevance of a specific project. If the audit result was positive, the institute should be given the freedom to run the project. The Bundestag’s new defense innovation and experimentation rapporteur could invite all institutes using this new flexible practice for an annual gathering to exchange lessons identified and discuss additional measures to advance cooperation.

#### Adopt a new innovation management method

Today’s approach to defense innovation in Germany is very much input focused. The debate about defense-relevant core technologies and how best to define them is a perfect illustration. Aside from the fact that the current definition is a mix of technologies, products, systems, and capabilities (Bundesregierung 2020, p. 3), the problem with a technology-driven approach to innovation is that it overlooks what technologies are expected to achieve. Truly innovative defense solutions only emerge when military end-users adopt and adapt to novel approaches thereby embedding technology in concepts, culture, organization, and operations (Borchert et al. 2021; Horowitz and Pinyck 2022; Raska 2016). Therefore, innovation management should become more outcome and impact focused.

To this purpose the MoD should adopt a Real-Option-based approach. Traditional valuation methods like capital budgeting with discounted cash flows cannot properly reflect strategic uncertainties or strategic surprises that characterize defense planning as the quantitative focus is ill-fitted to include qualitative aspects. The Real-Option method corrects this deficit by treating each position in a defense innovation portfolio like a financial option. This provides the innovation manger with much more flexibility in assessing long-term high-risk/high-benefit options at lower levels of maturity with short-term options disposing higher confidence levels as operational use cases and underpinning technologies are more mature. This method maximizes input and output:

“On the input side (innovation managers, force planners, and technology developers) can strive to maximize the contributions of individual technologies; on the output side they can set individual capability parameters and deduct from these parameters which technology – or combination of technologies – would generate maximum value” (Borchert et al. 2022, p. 35).

#### Advance defense sandboxing and strengthen defense test units

Sandboxes offer a “structured context for experimentation, enable where appropriate in a real-world environment the testing of innovative technologies, products or approaches” (Council of the European Union 2020, p. 4). Embraced by the 2019 German industrial policy (BMWi 2019b, p. 21) the German MoD should advance defense sandboxing to reduce the regulatory burden particularly for high-risk/high-benefit projects. The audit on current civilian admission criteria for defense products would be a good place to start as the findings could be turned into respective sandbox arrangements. This could, for example, entail laxer requirements on warranty obligations and product liabilities. In addition, defense sandboxing should modify the *Bonner Gewinnformel.* This profit tap effectively creates disincentives, in particular for growth-driven commercial companies working on emerging technologies that the MoD wants to integrate into its ecosystem.

Defense sandboxing should go hand in hand with a more vigorous use of existing military test and experimentation units to advance transformational change on the battlefield. To this purpose, the MoD should transform the existing test and experimentation unit of the Army in Munster into a fully-fledged military test lab for bi- and multinational partners. Similar initiatives for the navy, the air force, and the cyber and information command should follow. These labs will bring together operators, capability developers, industry, and research partners to work on future concepts, technologies, and products. Integrating foreign partners into these specific ecosystems could be especially beneficial as they would get in touch with German homologues at a very early stage. National labs could be systematically linked with binational military units such as the 1 German-Netherlands Corps and could be used to deepen cooperation with partners like the United Kingdom that envisages establishing similar units as part of the latest land industrial strategy (Ministry of Defence 2022, p. 22). Institutionalized channels from testing and experimentation into operations help overcome the “valley of death” that plagues the transfer of novel ideas from labs into reality. Furthermore, these test and experimentation units could become end users that participate in multinational projects under the current European Defense Fund and the future NATO defense innovation challenges, thus giving the Bundeswehr another lifeline to multinational defense innovation projects in return for operational insights.

#### Establish a Bundeswehr Digital Commons

The digitalization of the Bundeswehr is key to enhance existing capabilities, for example with autonomous systems or artificial intelligence. The challenge rests with setting up a digital environment that ignites dynamism and prevents monopolies. To this purpose we advocate creating a Bundeswehr Digital Commons (BDC) as a federated solution that includes existing digitalization initiatives to shape an environment in which software-augmented defense capabilities enhance the Bundeswehr’s versatility.

The BDC should particularly strive to establish a high-performance computing infrastructure to offer sophisticated digital simulation environments. This opens the door to model future defense capability requirements and defense solutions against highly agile and unconventional red teams commensurate with the ambition to become more daring. This synthetic simulation environment could also become a key asset in multinational cooperation.

Moreover, digital defense solutions penetrating every element of the sensor-operator-effector web are sovereign assets. As such, they need to be developed and operated in a trustworthy environment. This environment must reflect domain and mission-specific requirements and stimulate data sharing. Therefore, every user wanting to use data made available via the BDC needs to contribute data as well. This data-sharing dynamic must be driven by sovereign interest, not monetizable private interest, and comply with the highest safety and security standards. That’s why the Bundeswehr should maintain and operate the BDC while companies can offer applications, analytics, and specific software suits via specifically defined interfaces and protocols.

### Become a Defense Industrial Framework Nation

NATO introduced the framework nation principle at the 2014 Wales summit (NATO 2014). Berlin played a key role in developing the idea that one nation would provide the basic military building blocks to which partner nations could plug-in to advance multinational defense cooperation. The defense industry is a perfect means to create enduring lock-in effects that further Germany’s strategic interests, but it has hardly been used to this purpose. One of the problems stems from the fact that Germany increasingly lacks credibility in the eyes of its partners as the most recent controversy over defense supplies to Ukraine illustrates. Advancing the idea of a defense industrial framework nation can regain trust by clarifying ambitions with a view on partner interests, set up a government-to-government approach to facilitate cooperation, and stimulate knowledge transfer from partners to Germany.

#### Clarify your ambition

So far, the framework nation concept was equal to a “cost-sharing community”, which provided partners with the opportunity to piggy-back on Germany. This is far from matching the level of ambition needed to meet the current challenges. We see three options for Germany to raise its profile as a defense industrial framework nation:

- Option one builds on security of supply and is very close to the current approach. Security of supply has become a key issue given geoeconomic competition and the depletion of military stocks due to defense equipment aid for Ukraine. A defense industrial framework nation maximizing security of supply would ensure critical supplies for partners, as Rheinmetall is already providing ammunition for the Netherlands and the United Kingdom, for example. The Achilles’ heel of this option stems from Germany’s defense export regime as withholding deliveries to partners which would undermine the very security of supply this option is meant to create.
- Option two structures the defense industrial framework nation around technologies. Germany would strive to embed partners by focusing on developing specific technologies that are of mutual interest. Space technologies with commercial and defense applications, for example, could be leveraged to form clusters involving partners like Estonia, Finland, Luxemburg, the Netherlands, Norway, and Slovenia. These partners could operate as conceptual speed boots that challenge Germany’s incumbent space players to create a joint innovation dynamic.
- Option three is what we call the “rock the boat” approach. In this case Germany would take defense innovation very seriously and establish industrial clusters that focus on driving novel ideas. This approach would primarily emphasize the expertise of independent design bureaus, rapid prototyping, and experimentation companies as well as simulation experts. Germany would strive to assemble these partners in clusters to drive early-stage concepts and technology development thereby detaching the design phase of high-risk/high-benefit projects from large, traditional defense incumbents.

#### Set up a government-to-government framework

With the help of a government-to-government (G2G) framework governments purchase defense solutions from other governments rather than contracting defense companies directly. Governments rely on G2G to establish strategic relations with partners and benefit from financial terms and conditions defense suppliers offer their home nations. G2G can also provide direct channels of communication that the recipient can use in case of problems with suppliers from delivery nations. Germany engages in G2G but unlike the US, France, or the United Kingdom, a proper G2G framework is missing.

It is difficult to foresee how the German government wants to “strengthen defense industrial cooperation in Europe” (SPD, B90/Die Grünen & FDP, 2021, p. 149) in the absence of a G2G framework as projects under the European Defense Fund, for example, entail synchronizing capability and procurement requirements that depend on governmental defense planning. Defense planning, in turn, increasingly mirrors portfolio management with the need to balance capabilities, technologies, and partners against national and allied ambitions. In this context, a G2G concept needs to

- define more precisely how the German government wants to support international defense projects,
- clarify which national entities could be involved in multinational projects and what kind of support these entities can provide,
- establish and delineate individual and collective responsibilities among all relevant public and private sector partners,
- exemplify how ambassadors, military attaches, military technical attaches, and Bundeswehr units operating overseas can solidify and advance defense industrial cooperation with foreign partners,
- outline the terms of engagement companies need to respect when entering into agreements on foreign market access, defense industrial participation, as well as technology and know-how transfer.

Clarifying the different roles the German government can play to support defense cooperation is paramount. In this regard, we propose a three-layered approach:

- First, at the MoD-to-MoD level Germany can offer general training and education by inviting partners to join the German Command and Staff College and the Federal Academy for Security Policy; mission-specific training in Germany and in-country with a focus on properly using defense equipment; cooperate on capability planning and development as well as procurement including embedding German experts with partner institutions; offer partners access to dedicated German testing infrastructure including access to the proposed Bundeswehr Digital Commons; offer support on strategic project controlling including a “red phone” for access to the MoD in case of unresolved problems with German defense suppliers; joint exercises, missions, and operations; joint concept and design, R&T, and procurement projects including the set-up of collaborative institutions to support the respective projects.
- Second, at the MoD-to-interagency level G2G would strive to broaden the fields of cooperation by reaching out to other ministries. The German MoD can facilitate cooperation on strategic issues like emerging technologies and arms control, food security and human performance modification, energy security, maritime and port security, space cooperation, and cybersecurity. In addition, Germany can establish strategic risk dialogue with partners to share insights on issues of mutual interest. This dialogue can also serve to discuss the prospects of potential joint defense exports to third countries to identify possible red lines early on. At the interagency level, the government can also facilitate comprehensive financial packages to fund defense cooperation, including options to launch joint investment funds to advance collaborative projects in the fields mentioned above, as well as support for partners in international organizations.
- Finally, at the MoD-to-industry^3^ level the primary role is to build and sustain a coherent effort by structuring the team to meet the needs of the foreign G2G partner and advance German political and industrial interests. This coordination also entails match making in cooperation with foreign governments to introduce German business to foreign partners and vice versa.

#### Advance reverse empowerment

Germany gains valuable insights from cooperating with partners. While collaborative projects might explicitly focus on learning, we contend that material donations to empower partners have been strategically underexploited. Smartly played, donations to foreign partners open market segments for German defense companies abroad while raising the bar for competitors to enter the same field. Donations can also generate valuable business on maintenance, repair, and overhaul to extend the partnership. Finally, the current war in Ukraine creates a special situation in which German defense equipment undergoes the ultimate litmus test on the battlefield. This creates a unique opportunity for the Bundeswehr and the German defense industry to learn from Ukraine. The German MoD should provide institutional channels that can be used to infuse existing user clubs, formed among the operators of German defense systems, with Ukrainian insights as these could help adapt Bundeswehr concepts and improve defense products (Borchert & Schütz 2022).

### Promote defense industrial diplomacy with partners

“Diplomacy is about managing international relations to further national interests. Defense diplomacy is using defense assets to support diplomatic objectives and further defense interests” (Ministry of Defense 2014, p. 12). German defense companies should be interpreted as an important means of defense diplomacy as they have established a global presence that serves as an important transmission mechanism into foreign sovereign industries. For the time being, Germany has not strategically used its corporate footprint to advance defense interests abroad. Remedying the shortfall needs a clear understanding of the defense industrial portfolio. This provides the basis to leverage German defense companies as technology bridges to advance regional supply webs. In addition, defense industrial skills should be understood as an important currency to forge bilateral relationships.

#### Define the portfolio

Defense industrial diplomacy includes activities that German defense companies launch in support of corporate and sovereign German interests. The portfolio remains to be properly defined. For a start we suggest including the following tasks:

- Strategic advice on setting up and developing national defense industries
- Education and training of partner experts in Germany and in-country
- Support in establishing defense production facilities abroad
- Transfer and co-production of technology and know-how
- Promotion of STEM^4^ education in cooperation with German universities
- Joint approaches to advance defense supply chain agility and resilience, including joint supply chain risk assessments
- Joint stocks of critical assets and raw material with partners
- Joint export campaigns to third countries.

Defense industrial diplomacy is important, but also has limits. Defense is a sovereign industry. It needs to be treated with care as the unwanted proliferation of skills and technology creates negative side effects. That’s why the list of countries that benefit from German defense industrial diplomacy needs to be drafted with care. This will require guidelines that consider Germany’s sovereign and industrial interests, the partner’s regional security context, capability demands of the Bundeswehr and the foreign partner as well as the levels of maturity a foreign partner has achieved in terms of scientific, technical, and industrial proficiency.

#### Leverage German defense subsidiaries abroad as technology bridges to advance regional supply webs

Congruent industrial interests underpin successful defense cooperation; competing industrial interests constitute a stumbling block, even when political decision-makers are pushing for cooperation. We contend that – apart from German-Franco relations – Berlin does not yet use cross-national corporate defense ties to full extent.

We don’t argue that “the flag needs to follow trade”. Rather we argue that looking at complementary defense industrial portfolios whereby one nation can fill the gaps of another nation, would enable Berlin to develop defense industrial relations more strategically. Given the upcoming NATO membership of Sweden and Finland, northern Europe is a case in point. Germany and Norway (Kongsberg) cooperate on submarines, and Germany and Sweden (Saab) have a long-standing missile partnership. Theses relations could be complemented, for example, by cooperating with Finland on micro satellites (ICEYE), driving the development of unmanned systems (Milrem, DefSecIntel) and cyber defense (Cybernetica, Guardtime) with Estonia and engaging on next generation battle management systems with Denmark (Systematic).

KMW’s decision to take a stake in Estonia’s Milrem (KMW 2021) and Rheinmetall’s decision to have 150 out of 218 Lynx combat vehicles manufactured in Hungary (Reuters 2020) and establish a joint venture on defense digitalization with Hungarian companies 4iG and HM EI on defense digitalization (Rheinmetall 2022) is reminiscent of the integration of suppliers from Central and Eastern Europe into German automotive supply chains. This aspect is most important when considering security of supply amid growing geoeconomic tensions and the need to forward deploy military equipment and industrial capacities to frontline states. Engaging with partners on a regional basis could provide Germany a novel way to advance regional embeddedness.

This regional approach would move from supply chains to supply webs. Defense supply webs would provide more leeway by giving each partner a say through task delineated according to defense industrial maturity, existing capacities, and capability preferences. Given the strategic risk of depending in single sources of supply, supply webs would not follow a purely business logic but entail elements of resilience. Thus, supply webs would include multiple production entities, strategic stocks, and design entities that pool critical engineering expertise on a regional basis. Most importantly, supply webs could advance industrial interoperability to make sure the production of specific components can be easily reallocated among pre-defined facilities to provide instant support in case of interruptions.

Supply webs meant to advance regional defense cooperation also imply a high degree of responsibility. This not only affects Germany’s defense export policy but would also require Berlin to come up with novel approaches to stabilize supply chains under pressure, for example, by injecting liquidity to companies.

#### Consider defense skills a strategic asset

Sovereign defense industrial capacities require sovereign defense industrial skills. Because of the deteriorating geostrategic environment, governments take defense industrial skills seriously. Australia, for example, puts a major emphasis on shoring up the national defense industrial base and has promised “a large expansion of the Defence industry workforce over the next decade” (Department of Defence 2018, p. 55). The latest UK Land Industrial Strategy makes a comparable pledge and “expects businesses to demonstrate how they are investing in UK skills, infrastructure and innovation and will measure and monitor progress through contractual performance indicators” (Ministry of Defence 2022, p. 31).

Germany is well-known for its dual-track apprenticeship model. That is why a public-private approach to boost defense skills is a logical next step. Such a skilling initiative could make optimal use of expert defense workers that retire in Germany but might still want to continue sharing their experience with foreign partners. These experts and Bundeswehr veterans could be pooled and educated in train-the-trainer programs. Together with defense companies willing to include foreign workforce, they would form the nucleus of the defense skilling initiative. This initiative would be a prime instrument to fulfill defense offset obligations outside Europe as countries that strive to set up their own defense industry aspire to train their indigenous workforce.

### Solidify a True Public-Private Defense Partnership

The German MoD and the Association of the German Defense and Security Industry (BDSV 2022) have stepped up cooperation and established regular working formats to discuss policy issues.^5^ We see room for improvement with regard to tackling the impact of the EU’s social taxonomy on defense, the consequences of geoeconomic competition for defense companies, the need to readjust the local ecosystem, and competitive intelligence.

#### Clarify that the defense industry is ESG compatible

In the wake of the war in Ukraine and sanctions against Russia, political decision-makers and civil society have increased moral pressure on companies to stand by Western values and disengage from Russia. At the same time those companies that have for decades provided the products to defend these values are considered non-compliant with ESG and the draft social taxonomy of the European Commission. This attitude is inconsistent, deters investors, and puts defense companies at risk. More so, it undercuts the idea of NATO becoming a strategic investor in companies developing emerging technologies (Stoltenberg 2022) and undermines the ambition of the Bundeswehr Center for Digitalization and Technology Research to incubate startups that produce defense-relevant solutions.^6^

Even more problematic, the defense related ESG discussion is becoming a plaything of political interests. Bavaria, which prides itself in boosting and promoting its high-tech industry, is a case in point. State-owned financial institutes cancel ongoing business relations with defense companies (Waschinski 2021) and refrain from investing in high-technology companies that work for the Bundeswehr as this violates the very same institutions’ statutory investment principles.^7^ At the same time, however, Bavaria has tabled a motion for resolution in the Bundesrat, the constitutional body representing German *Länder,* that asks the Federal Government to make sure that the unfolding work on the social taxonomy shall not prevent the defense industry from having “access to sufficient funding opportunities” (Bundesrat 2022, p. 6).

How can you take the *Zeitenwende* seriously if political authorities discredit one of their own instruments of power by arguing that defense does not protect the constitutive values of Germany? This situation can be rectified in two ways.^8^ The boldest move is to simply exempt the defense industry from ESG requirements, for example, with a statement issued by the Minister of Finance and signed by all state-owned financial institutes at federal and *Länder* level. The other option is a conditional lift of ESG requirements by limiting non-compliance with ESG on those defense products that the German government considers unlawful under international law. Publishing a respective list would make it easy for all investors to verify if a defense company engages in such activities and decide if or not to invest.

#### Engage in technology road mapping with industry

In a structured dialogue the German MoD, defense companies and RTO are engaged in setting up defense R&T plans. Bundeswehr capability requirements and the defense budget inform this dialogue. As argued above, current geoeconomic competition is focusing on emerging technologies that are important for commercial and defense business models. That’s why the scope of existing talks should be broadened.

Future technology road mapping needs to consider the negative effects of countersanctions, informal market entry barriers, import and export limitations, foreign direct investments in Germany, implications of the proposed regional supply webs as well as requirements to redesign defense supply chains on German defense companies. This exercise also needs to include the interplay between individual technology plans, the strive for foreign market access, foreign market demands for technology transfer and localization. All these activities eat up existing competitive advantages. That’s why a consolidated overview is needed to identify looming risks for Germany’s very own technology ambitions.

Moreover, we see a growing need for the German MoD and the German Ministry of Education and Research to join forces. Together they need to raise awareness for the sovereign risks that civilian R&T cooperation with third countries can entail and come up with compulsory cooperation guidelines. Cooperation with China that benefits the People’s Liberation Army (PLA) is a case in point. Earlier this year, an investigation by CORRECTIV, Follow the Money and different newspapers (CORRECTIV 2022) revealed that several German universities cooperate with China’s Seven Sons, universities that work closely with the PLA. Among them are the RWTH Aachen, the Technical Universities in Darmstadt and Bremen and the University of Hamburg – all four institutions adhere to the *Zivilklausel* that prevents them from cooperating with the Bundeswehr.

#### Recalibrate competition vs consolidation

We have argued in section three that the German defense ecosystem is increasingly unbalanced. Part of the reason is that the relationship between demands for competition and consolidation are in limbo. The German government needs to be clear what it wants.

Throughout recent years, calls for increasing defense consolidation have been *en vogue*, particularly at the European level. Europe’s defense base has been described as balkanized with too many different systems in use that drive operational and maintenance costs (see for example Bundesregierung 2016, 129). But rising geoeconomic risks beg the question if efficiency (not effectiveness) is the right paradigm to approach this question. Consolidation of products and different product sub-types may be okay but are European nations sure about increasing consolidation of defense companies? This can endanger security of supply and the need to quickly remap up defense industrial production in war. Consolidation could also create increasingly attractive attack vectors for aggressors that only need to focus on a limited number of players.

Germany oscillates between “going European” as the default mode and a muted readiness to promote national solutions. There is no easy way out, but a more granular approach could help. Germany could embrace and even stimulate competition at the design stage of future defense solutions. The government could even invest money in making sure there are enough independent design entities that work with the big defense primes but do not belong to them. Once a winning design proposal has been selected, plans could be transferred to primes for production. Here, the level of competition would be lower, and this could provide an incentive to retain and pool critical skills.^9^

#### Advance competitive defense intelligence

Competitive intelligence provides insights on all aspects that shape the competitive environment of a company. With defense being a sovereign task, corporate insights are important but not enough. They must be blended with government expertise to frame a holistic picture of the challenges the German defense industry faces. Thus, competitive defense intelligence constitutes a joint task and supports strategic decision-making. Fusing public and private information is essential given the opaque nature of the defense business in many countries outside NATO and EU nations. To this purpose, local subsidiaries of German defense companies as well as defense and defense technical attachés need to be considered essential sensors that the competitive defense intelligence network needs to tap in. Competitive intelligence insights should become a regular issue of discussion when experts of the MoD and industry meet. A competitive intelligence cell, possibly attached to the BDSV with access to public and private stakeholders, could drive and coordinate the respective work.

## Conclusion

Making the Bundeswehr one of the most powerful armed forces (Freie Demokraten 2022) in Europe is a daunting task. Throwing € 100 billion at the defense establishment won’t do the trick, as the current defense ecosystem is unbalanced. For too long, the defense industry did indeed “walk alone,” to paraphrase Chancellor Scholz.^10^ Aside from procurement decisions, defense industrial issues have been anathema to German political discourse. Given the resurgence of a war in Europe and the prospect of enduring geoeconomic competition with strategic challengers, neglect of the German defense industry is short-sighted, dangerous, and unfit to advance German sovereign interests.

Defense is a sovereign industry that does not suffer from market failure, but policy failure. That is why the current funding boost must go hand in hand with a policy boost. We therefore present a defense industrial policy blueprint based on daringness as much needed prime ambition to repel a growing adversarial risk appetite. The blueprint expands on the idea of making Germany a defense industrial framework nation and suggests promoting defense industrial diplomacy with partners. Most importantly, the defense industrial policy framework must be underpinned by a true public-private partnership that needs to be reinvigorated and solidified. These are bold demands; implementing them constitutes a true *Zeitenwende.*

## Footnotes

1 The term was coined by chancellor Scholz on 27 February 2022 to describe his government’s response to Russian aggression in Ukraine.

2 For more, see: Initiative Hochschulen für den Frieden (o.J.).

3 Here, industry refers to traditional defense suppliers as well as commercial businesses from adjacent industry sectors like digitalization, energy, manufacturing, materials, and transportation to name but a few examples.

4 STEM stands for science, technology, engineering, mathematics.

5 For more on these working formats, see BDSV (2022).

6 For more on this, see dtec.bw (2022).

7 Based on background information provided in February and March 2022.

8 Recently, the European Commission has invited the EU member states as shareholders of the European Investment Bank to “assess, whether, under present market and geopolitical circumstances, it should extend its support to such defence-related industrial projects, by adapting, if necessary, its lending policy.” (European Commission 2022, p. 13).

9 The latest UK Land Industrial Strategy includes a similar proposal that builds on a “shift in policy away from global competition by default” (Ministry of Defense 2022, p 10).

10 On July 22, 2022, Chancellor Scholz promised further relief to German citizens that need to cope increasing energy costs in the wake of Russia reducing gas supplies to Germany. He said “You’ll never walk alone” to illustrate that nobody would be left behind (Sozialdemokratische Partei Deutschlands 2022).
